# Stable preservation and recovery of methylation marks from FTA elute cards in species with nucleated red blood cells using a customized DNA extraction method

**DOI:** 10.1371/journal.pone.0329019

**Published:** 2025-07-31

**Authors:** Livia Gerber, Sarah L. Whiteley, Erin E. Hahn, Clare E. Holleley

**Affiliations:** 1 Australian National Wildlife Collection CSIRO, National Research Collections Australia, Black Mountain, ACT, Australia; 2 Institute for Applied Ecology, University of Canberra, Bruce, ACT, Australia; University of Saskatchewan, CANADA

## Abstract

Specialized chemically-coated paper cards, such as Flinders Technology Associates (FTA) cards, provide simple and reliable storage of nucleic acids by protecting DNA from degradation. Owed to their simplicity, FTA cards are widely used in clinical testing, forensic science and specimen archives. Originally developed for PCR-based applications that only require short DNA fragments, FTA cards are now being explored as an avenue for whole-genome and epigenetic sequencing applications. FTA cards and their corresponding DNA extraction protocols have not kept pace with advances in sequencing technologies. Because the initial protocols developed for FTA cards were geared towards applications using PCR amplification of short fragments, they typically yield low molecular weight DNA. This issue is particularly pronounced for FTA elute cards where heat-based elution at 95°C leads to DNA denaturation and fragmentation. Isolation of DNA from nucleated blood deposited onto FTA elute cards poses an additional challenge when compared to FTA classic cards, because hemoglobin is irreversibly bound to the card matrix, making the majority of DNA present in nucleated blood inaccessible. Here, we describe an easy, fast, and inexpensive protocol to extract high molecular weight DNA (>10 kb) of nucleated blood stored on FTA elute cards suitable for most genomic library preparations including those that interrogate DNA methylation. Our protocol yields a 14-fold increase in yield compared to numerous alternatives. Using our protocol, we demonstrate that high molecular weight DNA can still be extracted even after storage at ambient temperature for over a decade. Moreover, we show that DNA methylation marks are preserved on FTA elute cards, broadening the utility of FTA elute cards. This opens possibilities for (epi-)genomic studies using historical samples and enabling specimen collection where access to chemicals or cryogenic storage is limited – reducing project costs and extending collection opportunities into remote areas.

## Introduction

Flinders Technology Associates (FTA) cards are a form of specialized chemically coated paper cards that protect DNA from degradation. Widely used in clinical testing [1], biobanks [2,3], forensic science [4,5] and specimen archives [6], FTA cards are a simple option for sampling and storing biological samples for downstream DNA extraction. Once a sample is applied, no additional equipment or temperature control is required, making FTA cards a small, lightweight, and self-sufficient specimen storage medium. This makes them a desirable choice to store samples for large-scale or high throughput projects, such as routine medical screenings [7] or for collecting biological materials from wild animals or plants in remote areas [6,8].

Two types of Whatman FTA cards exist: classic and elute. The two card types share the ease of sample collection and storage but use different proprietary DNA-binding chemical constituents and thus have different mechanisms of action and require different downstream processing. Specifically, the two card types necessitate different DNA extraction protocols. FTA classic cards use a preservation approach that permanently binds nucleic acids to the card and are typically processed via direct DNA amplification directly from a small card punch (<1mm) after a washing step. This extraction procedure is commonly referred to as ‘punch-in’ method [9]. In contrast to the classic cards, FTA elute cards are designed to enable DNA elution from the cards using a ‘boiling’ protocol, which is the manufacturer’s recommended protocol. This simple DNA extraction involves washing the FTA elute card punch (1–8 mm) and then incubating in sterile water or QIAcard FTA Elute Buffer (Qiagen WB120100) for up to 20–30 min at 95°C [10]. High temperature treatment denatures and dissociates the DNA from the fibers of the FTA elute card, releasing it into the water, but it also shears DNA. Proteins and PCR inhibitors remain bound to the FTA elute card matrix so that no further cleanup is necessary before PCR.

Because current extraction methods for both classic and elute FTA cards produce highly fragmented DNA, their routine use is restricted to traditional short-read and amplicon sequencing techniques, such as microsatellite and mitochondrial genotyping [6,11,12]. Scientists in the medical field have started to use alternative protocols to extract longer DNA fragments from FTA cards used for newborn screening [13,14], unlocking a vast number of archival samples for whole-genome and DNA methylation studies [15]. High molecular weight (HMW) DNA extraction protocols from FTA cards often mimic those developed for other tissue sources and include organic phenol-chloroform extraction and costly commercial kits [16]. However, now that (epi-)genome-wide data can be generated at low costs, there is increasing demand for HMW DNA from FTA cards from a broader range of species, beyond human and mammalian model systems [6,17,18].

Many vertebrate species (all birds, reptiles, and fish) have nucleated red blood cells, which adds a unique complication to the use of FTA cards compared to the more mainstream applications using non-nucleated mammalian blood. DNA extraction from nucleated blood spotted on FTA elute cards poses a technical challenge when compared to FTA classic cards because hemoglobin, a component of red blood cells and a known PCR inhibitor, is irreversibly bound to the FTA elute card matrix. Although this propensity of FTA elute cards is useful to avoid hemoglobin contamination in the isolated DNA when the boiling method is used, it significantly reduces DNA recovery from nucleated blood if the contents of red blood cells remain trapped on the card. To the best of our knowledge, no protocol for extracting HMW DNA suitable for genomic applications has been developed for nucleated blood stored on FTA elute cards, or even FTA elute cards in general. This is because (epi-)genomic studies either used FTA classic cards [17–19] or do not specify which FTA card type was used [13–15]. To make FTA elute cards more broadly accessible for (epi-)genomic applications, we developed a novel protocol permitting the extraction of HMW DNA fragments (>10 kb) from nucleated blood stored on FTA elute cards. To showcase the usability of this protocol for short-read sequencing and DNA methylation interrogation, we demonstrate that read count across read length and DNA methylation levels at CpG sites is preserved. This is achieved by sequencing nucleated blood collected of bearded dragons (*Pogona vitticeps*) stored on FTA elute cards and a control of frozen blood.

## Materials and methods

The protocol described in this peer-reviewed article is published on protocols.io (dx.doi.org/10.17504/protocols.io.36wgqnmq5gk5/v1) and is included for printing as S1 File.

### Sample collection and storage

To demonstrate the efficiency of our protocol, we used blood collected from a captive colony of central bearded dragons (*Pogona vitticeps*) held at the University of Canberra where FTA elute cards have been used to support genetic sex identification for more than a decade. Bearded dragons are an emerging model for genomics, reproductive and developmental research [20–24]. After blood application, FTA elute cards were left to dry and then stored at ambient temperature for 1–11 years. We expect our protocol to generate similar results as described here on samples collected and stored in a similar fashion. While we only tested our protocol on nucleated blood, we presume it would work with other sample types stored on FTA elute cards.

### DNA extraction from frozen blood used as control

**Fig 1:**
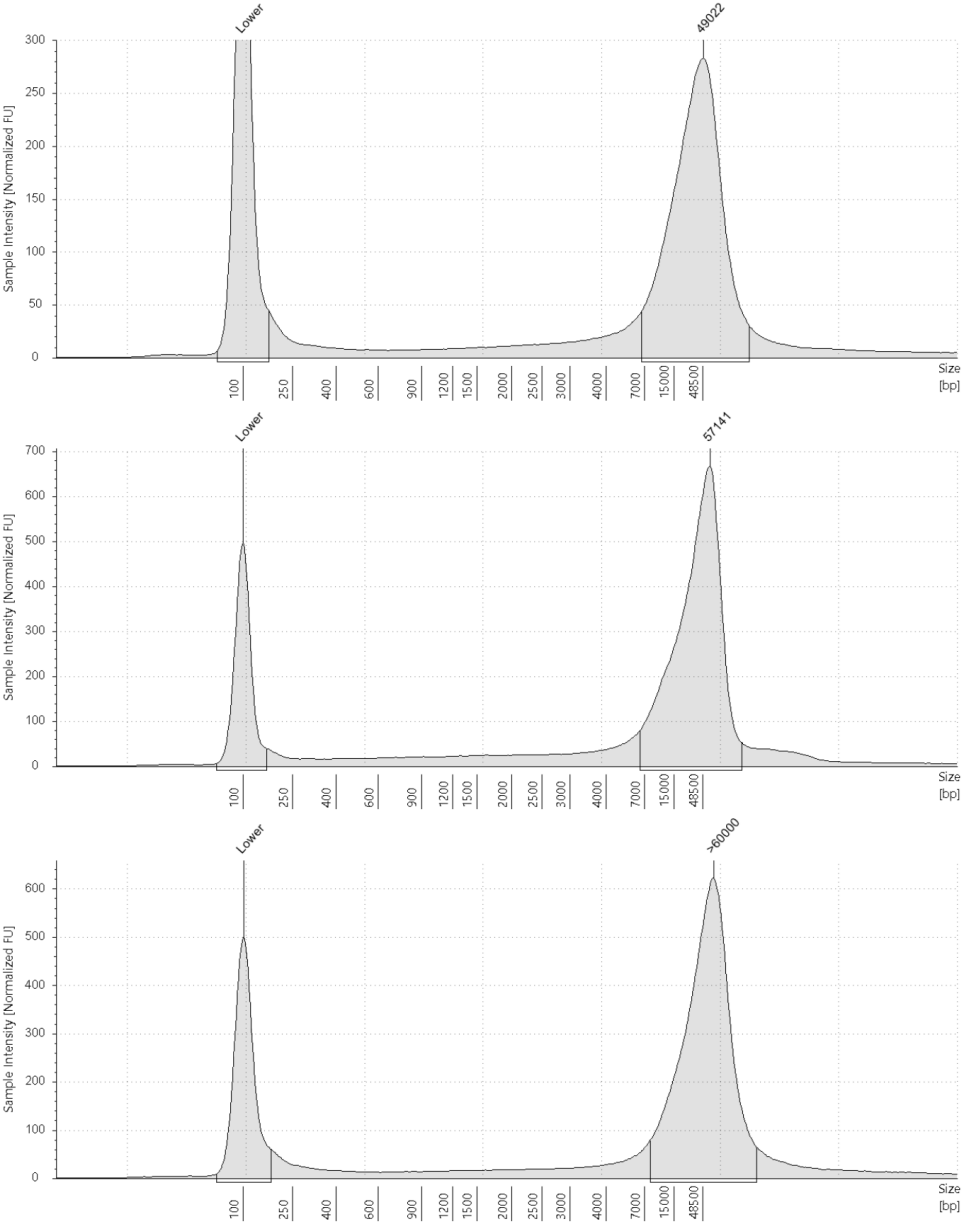
TapeStation reports of DNA extracts from frozen blood. TapeStation reports confirming high molecular weight of DNA extracts from frozen blood.

Frozen blood samples were extracted with a Puregene Tissue Kit (Qiagen 158063). We chose the tissue kit because the Puregene Blood Kit lyses and removes red blood cells in one of the first steps which is undesirable when working with nucleated blood. Before adding ~8 µl of blood per sample to the Cell Lysis Solution, we thawed the blood samples in a 37°C water bath. We followed the manufacturer’s instructions minus the initial grinding step. The Puregene kit yields DNA of high molecular weight which we have confirmed by running three randomly chosen samples on the TapeStation (Fig 1). Mean yield of DNA extracted from frozen samples was 8,701 ± 7971 ng (quantified using a Qubit 4 Fluorometer with the 1x dsDNA Broad Range Assay kit (Q33267)). The range is variable because the blood was frozen without additives which resulted in blood clots that made pipetting accurate starting volumes difficult.

### RRBS sequencing and bioinformatic analyses

To demonstrate the utility of our protocol for short read (epi-)genomic sequencing, we used the HMW DNA extracted by following our novel protocol to generate DNA methylation data with a reduced representation bisulfite sequencing (RRBS) approach. For library preparation, we used the Ovation RRBS Methyl-Seq system from NuGEN followed by paired-end 150 bp Illumina sequencing on a NovaSeq X. We sequenced 61 extracts from FTA elute cards using the protocol described herein. As a positive control, we also sequenced 25 DNA extracts from snap frozen reptile blood that was not stored on FTA elute cards.

We followed NuGEN’s analysis guide for adaptor and quality trimming of the raw paired-end reads (https://github.com/nugentechnologies/NuMetRRBS). We aligned the trimmed sequences to the bearded dragon genome (GCA_900067755.1) using Bismark [25]. To increase the mapping efficiency, we modified the maximum insert size to 700 bp and applied more relaxed alignment criteria for R2 (--score_min L,0,-0.4). We used Bismark for methylation calling using default settings but ignoring the first 3 bp on R1 and the first 2 bp on R2, as customarily done with RRBS data [26]. Finally, we filtered the methylation data to achieve a minimum read depth of 10x per locus for each sample using min_coverage in methylkit [27] in R v4.1.3 [28]. This last step permitted us to infer if methylation levels were different between the data generated from DNA extracts from FTA cards and such from frozen samples.

### Statistical analyses

All statistical analyses were carried out in R v4.4.0. Linear Mixed Models were run using lme4 [29].

### Ethics declarations

University of Canberra Animal Ethics approval no. CEAE 17−08 and CSIRO AEC 2024−12.

## Expected results

### Expected DNA quality, quantity, and molecular weight

We successfully used our protocol to extract pure HMW (>10 kb) genomic DNA from FTA elute cards spotted with nucleated blood. Post clean up, we saw no evidence of either protein or salt residue contamination (Nanodrop ratios: 260/230 mean = 2.3 ± 0.06; 260/280 mean = 1.9 ± 0.04). From two 3 mm punches per card, we obtained an average of 2538 ng ± 1589 (min = 420 ng, max = 8075 ng, N = 40) total DNA, which is sufficient for most genomic library preparations including reduced representation bisulfite sequencing (RRBS), whole genome bisulfite sequencing (WGBS), and other short read sequencing applications. The variability (420 ng to 8075 ng) arises because not all 3 mm punches on an FTA card contain equal numbers of blood cells. Some punches are more saturated with blood than others, resulting in higher genomic DNA yields where the saturation is greater (see photo S2 File). The disparities in blood saturation levels among the punches account for the extensive range observed between the minimum and maximum yields. Owing to the high yield of DNA in nucleated blood, a single 3 mm punch may be sufficient for many genomic applications in cases where sample availability is limited.

**Fig 2:**
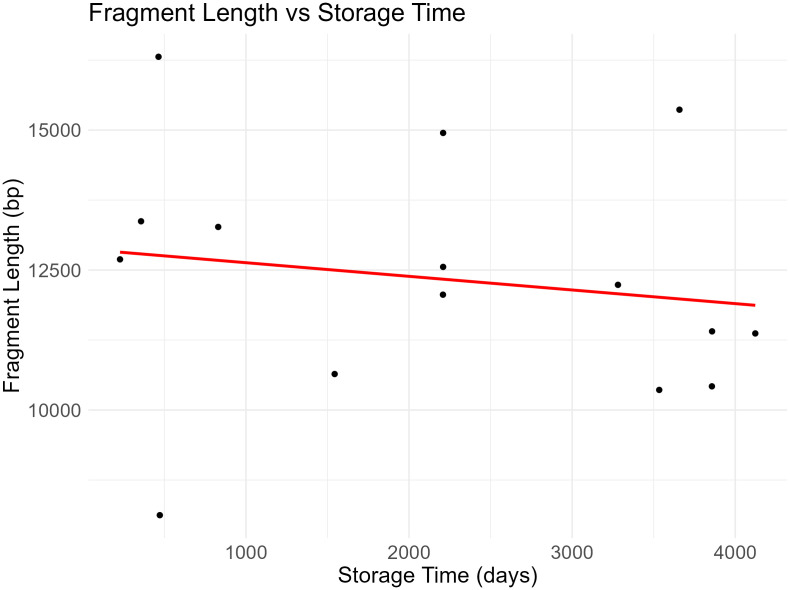
Relationship between storage time of nucleated blood samples on FTA elute cards and DNA fragment length. There is a non-significant negative influence of storage time on fragment length (N = 15, p = 0.221).

**Fig 3:**
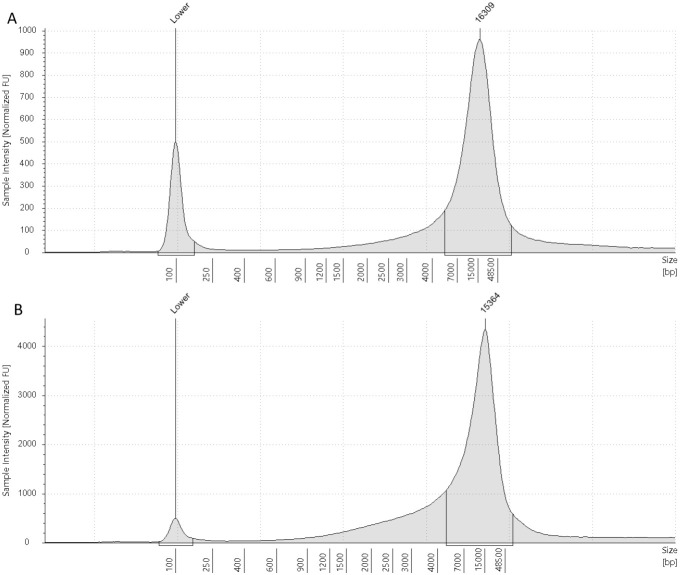
TapeStation reports of DNA extracts from FTA elute cards. TapeStation reports of DNA extracts from FTA elute cards stored for one year (A) and ten years (B) at room temperature. The mean fragment sizes are 16 and 15 kb.

FTA elute card extracts run on the TapeStation 4150 (Agilent) with the genomic ScreenTape indicated average fragment lengths between 10–15 kb. Like in formalin-preserved archival samples [30], there was no evidence of DNA degradation correlating with increased storage time (Spearman’s rho = −0.336, p = 0.221, N = 15, Fig 2), suggesting that nucleated blood samples applied to FTA elute cards can be stored at room temperature for well over a decade (Fig 3). This result contrasts with a recent study using FTA classic cards spotted with non-nucleated mammalian that did observe a decrease in DNA fragment size with storage time, despite the cards being stored frozen [18]. It is unclear if this discrepancy is due to the type of FTA card (elute versus classic), the storage conditions (ambient versus frozen) or the sample type (non-nucleated versus nucleated).

### Comparison of mean read count and DNA methylation level between reads derived from FTA elute cards and frozen blood

**Fig 4:**
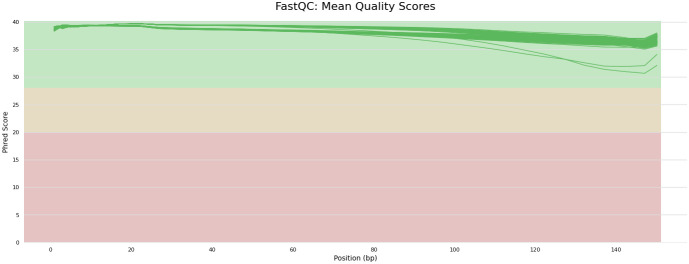
FastQC plot of RRBS reads. FastQC plot of filtered and trimmed samples from frozen blood (N = 25) and blood stored on FTA elute cards (N = 61). Reads of both sample types were of high quality (Phred score > 30).

Post trimming, sequences derived from samples stored on FTA elute cards and frozen blood were of high quality (Phred scores > 30 for all samples, Fig 4).

**Fig 5:**
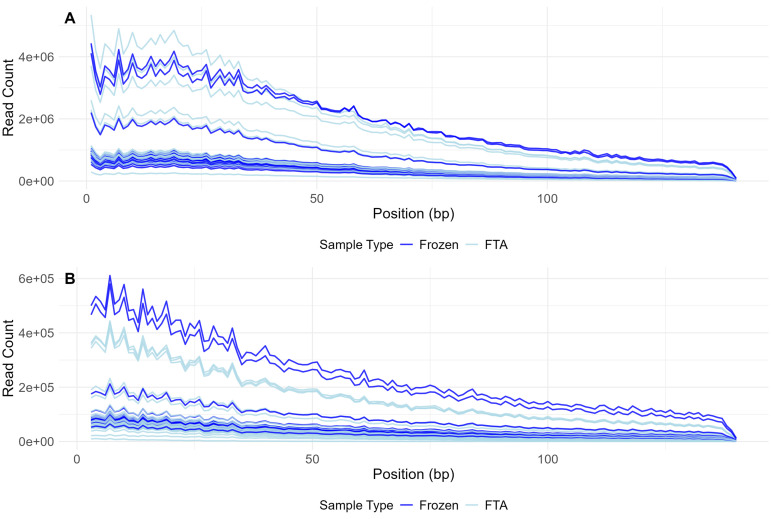
Read count across read position. Read count across read position of R1 (A) and R2 (B) of all 86 samples (25 frozen blood and 61 FTA elute cards). Please note that the maximum value of the y-axis is different between the two panels.

We observed no difference in read count across read position between sequences derived from FTA elute cards and frozen blood in R1 (linear mixed model, read count ~ sample type + (1|sample R1/R2), β = −62246, SE = 88970, t(84) = −0.700, p = 0.486, Fig 5A). On R2 in contrast, the read counts of FTA elute card samples were significantly lower across read position (linear mixed model, β = −18561, SE = 9311, t(84) = −1.994, p = 0.049, Fig 5B). We explain this with the shorter fragment length of DNA extracts from FTA elute cards (10–15 kb) when compared to frozen blood (> 50 kb). This may lead to fewer fragments of twice the read length (2 x 150 bp) generated from FTA elute samples and thus, a larger proportion of insufficient template to fully sequence R2 when compared to frozen blood.

**Fig 6:**
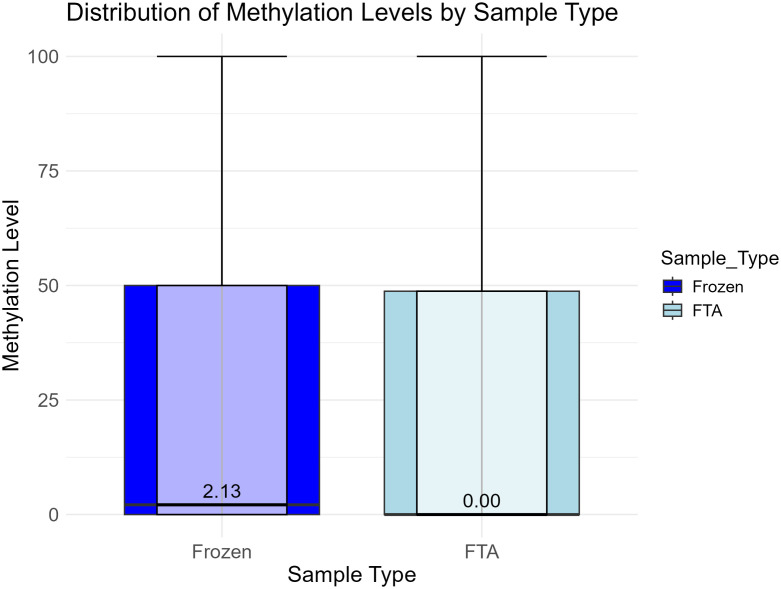
Distribution of methylation levels by sample type. The boxplot shows the distribution of DNA methylation levels in samples collected using FTA cards (N = 61) and frozen samples (N = 25). Each box represents the interquartile range (IQR) with the median indicated by the horizontal black line inside the box and the median values in numbers just above the line. The whiskers extend to the most extreme data points within 1.5 times the IQR from the quartiles. FTA card samples are coloured in light blue and frozen samples in dark blue. A Welch’s t-test showed no statistically significant difference between the two groups (p > 0.05).

The mean methylation level for FTA elute samples (N = 61, mean ± SD = 24.1569 ± 1.2645) was lower than for frozen samples (N = 25, mean ± SD = 25.1535 ± 2.8003). However, this difference was not statistically significant (Welch’s unpaired t-test: t(28) = −1.709, p = 0.098, d = 0.459, Fig 6) and may be caused by biological differences because the samples were collected from different individuals at various times during their life. A future study is needed to infer if storage on FTA elute cards cause subtle changes in DNA methylation. This could be done by analyzing matched samples where half of a sample was stored on an FTA elute card and the other half was snap-frozen as previously evaluated for FTA classic cards where matched samples were highly correlated [15].

### Alternative unsuccessful DNA extraction protocols trialed

Along with the successful DNA extraction described here, we also report a number of approaches that do not yield DNA of sufficient quality and quantity for downstream applications. The following extraction approaches are not suitable for DNA extraction of FTA elute cards for nucleated blood samples: Puregene Blood and Tissue Kit (Qiagen 158063), DNeasy Blood and Tissue Kit (69506), ChargeSwitch Forensic DNA extraction (ThermoFisher CS11200), Quick-DNA HMW MagBead (Zymo Research D6060), Monarch HMW DNA Extraction Kit (New England Biolabs T3060), FTA elute protocol (Qiagen WB120100), organic extraction using phenol chloroform [31] and a modified CTAB extraction [32]. All protocols were carried out according to the manufacturer’s instructions except the FTA elute protocol in which we omitted the 95°C Proteinase K inactivation to avoid denaturation of DNA strands. Therefore, simply omitting the heating step of the current FTA elute card manufacturer’s protocol does not yield sufficient DNA for (epi-)genomic analyses. The approaches listed above all recovered only small amounts of low molecular weight DNA (mean yield < 150 ng, Table 1) and thus yield around fourteen times less DNA compared to the comparatively simple DNA extraction method presented here (mean yield >2000 ng). We selected the trial kits based on their proven effectiveness for DNA extraction from reptile blood and tissue samples. Given that FTA elute cards irreversibly bind hemoglobin, we hypothesize this prevents red blood cell lysis, thus, significantly reducing DNA yield from nucleated blood samples. Our protocol uses physical maceration of the cards to free red blood cells from the FTA elute card matrix and make them available for lysis. We note that none of the unsuccessful protocols included a card maceration step (as per manufacturer’s instructions). Future investigations are needed to test if adding a maceration step could increase DNA yield. We emphasize the importance of understanding the properties of FTA cards with different chemistries where extraction methods are not necessarily interchangeable. For example, DNA from nucleated blood can be extracted successfully from FTA classic cards (as opposed to elute cards), using a commercial kit and without maceration [17,19].

**Table 1:** Alternative DNA extraction protocols trialed.

Protocol	Concentration of recovered DNA	Total DNA recovered from two 3 mm punches
**Recommended protocol**		
Gerber et al – this study	84.6 ng/ul eluted in 30 µl (N = 40)	**2538 ng**
**Not recommended protocols**		
Puregene	2.5 ng/µl eluted in 50 µl (N = 6)	123 ng
DNeasy	3.1 ng/µl eluted in 50 µl (N = 1)	155 ng
ChargeSwitch	1.9ng/µl in 150 µl N = 2	285 ng
Monarch	2.8 ng/µl eluted in 50 µl (N = 2)	140 ng
Quick DNA MagBead	Below detection (N = 1)	NA
FTA elute	2.2 ng/µl in 30 µl (N = 1)	67 ng
Phenol Chloroform	Below detection limit in 50 µl (N = 2)	NA
CTAB	3.9 ng/µl In 30 ul (N = 2)	117 ng

Overview of all alternative protocols trialed and their DNA yield quantified using a Qubit 4 Fluorometer with the 1x dsDNA Broad Range Assay kit (Q33267).

## Conclusions

Our findings confirm that as on FTA classic cards [15], DNA methylation marks are preserved on FTA elute cards for a prolonged period of time, making them an appropriate storage media for use in (epi-)genomic studies. Our protocol is a cost-effective and simple method for HMW DNA extraction from nucleated blood stored on FTA elute cards. The gentle and effective protocol described here yields HMW DNA suitable for genomic and DNA methylation studies requiring DNA up to 15kb. It remains to be tested if our DNA extraction protocol can also be used to extract DNA from other sources including non-nucleated blood (e.g., mammalian species). We expect non-nucleated applications will require a larger number of punches to obtain DNA yields reported here. Our protocol enables more researchers to collect and store their samples on FTA elute cards, especially in areas where access to chemicals or cryogenic storage is limited, such as in remote areas, developing countries, fishing vessels, and collections and museums with limited options for refrigerating or freezing samples.

## Supporting information

S1 File Step-by-step protocol, also available on protocols.io. (PDF)

S2 File Photo of FTA elute cards containing various amounts of blood. (JPG)

S3 File Raw uncropped images of TapeStation results as.png (ZIP)

S4 File Full uncropped multiqc report. (HTML)
