# Supplementary material for: Stable preservation and recovery of methylation marks from FTA elute cards in species with nucleated red blood cells using a customized DNA extraction method
Source: PLoS One. 2025 Jul 31;20(7):e0329019. doi: 10.1371/journal.pone.0329019 (PMC12312899; doi:10.1371/journal.pone.0329019)
Supplement: S1 File — (PDF) [file pone.0329019.s001.pdf]

Jul 14, 2025

gDNA extraction of nucleated blood from FTA elute cards

DOI
dx.doi.org/10.17504/protocols.io.36wgqnmq5gk5/v1

Livia Gerber[1], Sarah Whiteley[2], Erin E Hahn[1], Clare Holleley[1]
[1]CSIRO; [2]University of Canberra

Livia Gerber
CSIRO

OPEN ACCESS

DOI: dx.doi.org/10.17504/protocols.io.36wgqnmq5gk5/v1

Protocol Citation: Livia Gerber, Sarah Whiteley, Erin E Hahn, Clare Holleley 2025. gDNA extraction of nucleated blood from FTA elute cards. **protocols.io** https://dx.doi.org/10.17504/protocols.io.36wgqnmq5gk5/v1

License: This is an open access protocol distributed under the terms of the **Creative Commons Attribution License**, which permits unrestricted use, distribution, and reproduction in any medium, provided the original author and source are credited

Protocol status: Working
We use this protocol and it's working

Created: July 16, 2024

Last Modified: July 14, 2025

Protocol Integer ID: 103466

Keywords: FTA card, Nucleated red blood cell, Archival DNA, FTA elute card, genomic DNA, DNA methylation, gdna extraction of nucleated blood, corresponding dna extraction protocol, dna extraction, cards suitable for most genomic library preparation, low molecular weight dna, gdna extraction, short dna fragment, protecting dna, high molecular weight dna, isolation of dna, sequencing technology, reliable storage of nucleic acid, molecular weight dna, dna, dna from degradation, majority of dna present, dna present, dna denaturation, nucleated blood, dna methylation, most genomic library preparation, sequencing application, fta card, nucleic acid, pcr amplification of short fragment, dna methylation mark, fta classic card, fta elute cards specialized, coated paper card, pcr, paper card, card matrix, card, using pcr amplification, specimen archive

Funders Acknowledgements:
CSIRO Future Science Platform
Grant ID: R-19568

Abstract

Specialized chemically-coated paper cards, such as Flinders Technology Associates (FTA) cards, provide simple and reliable storage of nucleic acids by protecting DNA from degradation. Owed to their simplicity, FTA cards are widely used in clinical testing, forensic science and specimen archives. Originally developed for PCR-based applications that only require short DNA fragments, FTA cards are now being explored as an avenue for whole-genome and epigenetic sequencing applications. FTA cards and their corresponding DNA extraction protocols have not kept pace with advances in sequencing technologies. Because the initial protocols developed for FTA cards were geared towards applications using PCR amplification of short fragments, they typically yield low molecular weight DNA. This issue is particularly pronounced for FTA elute cards where heat-based elution at 95°C leads to DNA denaturation and fragmentation. Isolation of DNA from nucleated blood deposited onto FTA elute cards poses an additional challenge when compared to FTA classic cards, because hemoglobin is irreversibly bound to the card matrix, making the majority of DNA present in nucleated blood inaccessible. Here, we describe an easy, fast, and inexpensive protocol to extract high molecular weight DNA (>10 kb) of nucleated blood stored on FTA elute cards suitable for most genomic library preparations including those that interrogate DNA methylation. Our protocol yields a 14-fold increase in yield compared to numerous alternatives. Using our protocol, we demonstrate that high molecular weight DNA can still be extracted even after storage at ambient temperature for over a decade. Moreover, we show that DNA methylation marks are preserved on FTA elute cards, broadening the utility of FTA elute cards. This opens possibilities for (epi-)genomic studies using historical samples and enabling specimen collection where access to chemicals or cryogenic storage is limited – reducing project costs and extending collection opportunities into remote areas.

Guidelines

The processing times for each step are indicative for a sample size of eight.

Materials

Equipment and consumables
FTA card punchers
2 ml safe-lock tubes and 1.5 or 2 ml tubes suitable for storage
Tips
High quality 5 mm stainless steel beads
Bunsen burner
Thermal shaker
Qubit or another reliable method for DNA quantification

Reagents
Ethanol for flaming
low TE buffer (10 mM Tris, 0.1 mM EDTA)
ddH_2O
Proteinase K (20 mg/ml)
RNAse A (100 mg/ml)
Tris-Cl pH 8.5
Genomic DNA Clean & Concentrator-10 (Zymo D4010/4011)

Safety warnings

Ensure familiarisation with chemicals and choose appropriate PPE before commencing this protocol.

Ethics statement

University of Canberra Animal Ethics approval no. CEAE 17-08
CSIRO AEC 2024-12

Before start

- Label a 2 ml tube and a final storage tube (1.5 or 2 ml) for each sample.
- Sterilise the same number of 5 mm stainless steel beads as samples plus some spares.
- Set thermal shaker to 56°C.

Sample preparation

1h 10m

1 Punch two 3 mm punches from an FTA elute card into a 2 ml tube.

30m

Note

Sterilise puncher (e.g. flaming) between samples to avoid cross-contamination.

2 Wash punches twice with low TE and once with ddH_2O. Each wash step is carried out in the 2 ml tube and consists of adding 500 µl of either TE (first two washes) or ddH_2O (third wash), followed by vigorously vortexing for 5 s and subsequent removal of all 500 µl of wash solution but not punches.

40m

Note

If more than eight samples are processed, carry out the washing step in batches of eight samples to avoid prolonged exposure of the punches to TE or ddH_2O. Prolonged exposure negatively affects DNA yield.

Proteinase K and RNA digest

18h 30m

3 Add 200 µl TE, 10 µl Proteinase K and a stainless bead to each sample.

10m

Note

- The addition of the stainless steel bead is crucial. If no bead is added, DNA yield is significantly reduced.
- It is crucial to add a high quality stainless bead (e.g. Qiagen 5 mm stainless steel beads 69989). Low quality beads may rust, resulting in DNA degradation.

4 Digest overnight at 56°C on a thermal shaker. Set shaker to 500 rpm.

16h

5 Remove samples from the thermal shaker and pulse-vortex each sample for 1 min. Add 10 µl Proteinase K to each sample. 15m

6 Place samples in the heat block and incubate 1-2 hours at 56°C, shaking at 800 rpm. 1h 30m

Expected result

The punches should no longer be intact after this step. If this is not the case, continue incubation and pulse-vortex the samples repeatedly.

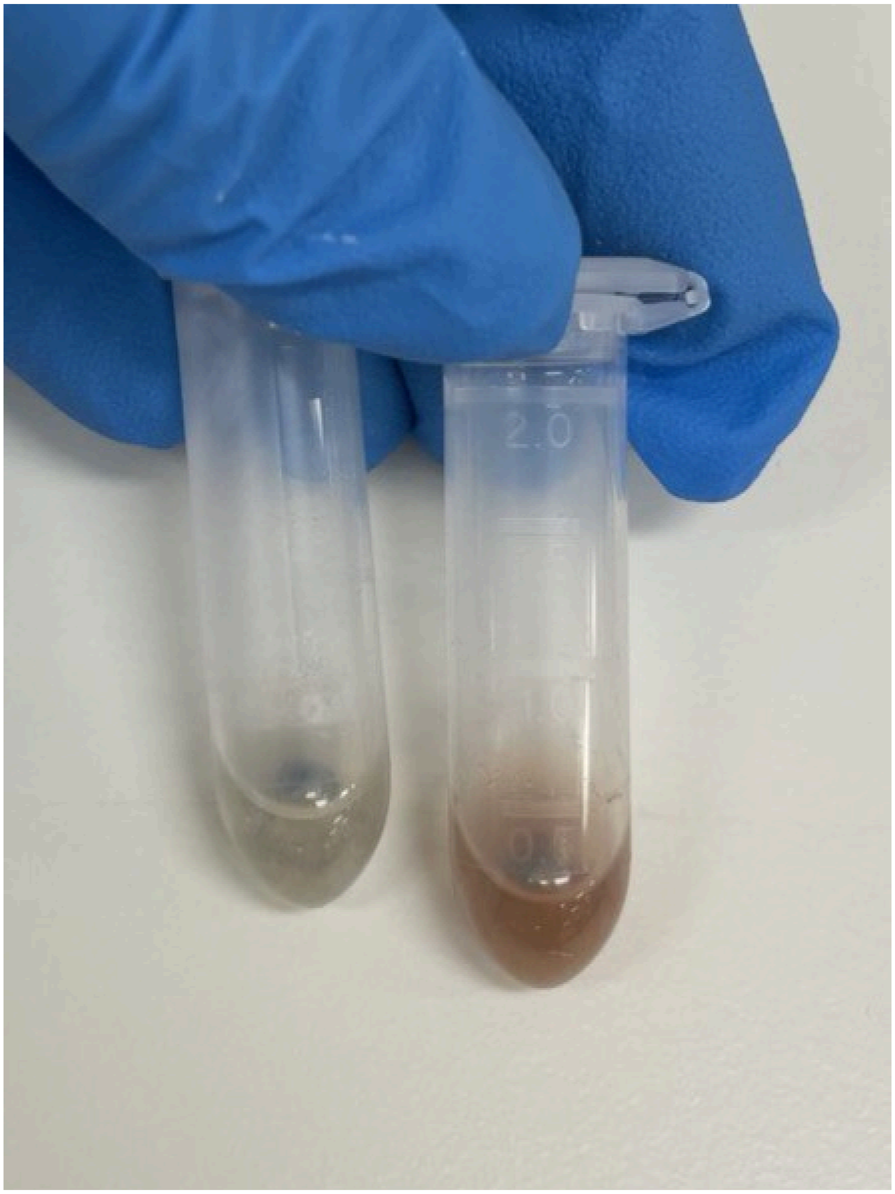

Two completely lysed samples. The colour of the sample depends on the amount of blood on the punches. Samples with higher amounts appear more red compared to samples with smaller amounts of blood which appear grey.

7 Cool samples to room temperature. Add 1.5 µl RNAse A to each sample and digest for 30 minutes at 37°C. Shake at 500 rpm during RNA digest to break punches down even further. 35m

Extraction clean-up

1h 15m

8 Clean extract using Zymo genomic clean & concentrator according to manufacturer's instructions. We added 800 µl ChIp DNA binding buffer to the punch lysate and included a dry spin for 2 minutes after the second wash with wash buffer. We eluted the clean DNA in 25 µl heated (60°C) Tris-Cl (pH 8.5) to maximise DNA in our eluate. 30m

Note

It may be possible to clean up the samples using SPRI beads. However, in a trial using SPRI beads DNA recovery was lower compared to the Zymo kit.

Quantification and quality check

45m

9 Quantification:
We quantified the purified DNA extracts with a Qubit. We recommend the dsDNA BR over the dsDNA HS assay because some extracts may be out of range (too high) for the HS kit. 45m

Purity assessment:
Purity of extracts can be assessed using a Nanodrop spectrophotometer or a similar device.

Fragment length estimation:
To evaluate fragment length, we used a TapeStation 4150.

Expected result

Using an elution volume of 25 µl, we obtained a mean DNA concentration of 101.25 ng/µl (lowest extract 17 ng/µl, highest 323 ng/µl) when measured using a Qubit 4 and the dsDNA BR assay.

Our extracts were of high purity (Nanodrop ratios: 260/230 mean = 2.3 ± 0.06; 260/280 mean = 1.9 ± 0.04).

From two 3mm punches containing nucleated blood, the overall yield is therefore approximately 2.5 µg with with an expected fragment size between 10 - 14 kb (measured on a TapeStation 4150 with the genomic DNA ScreenTape).
