# Supplementary figures and images for: Stable preservation and recovery of methylation marks from FTA elute cards in species with nucleated red blood cells using a customized DNA extraction method
Source: PLoS One. 2025 Jul 31;20(7):e0329019. doi: 10.1371/journal.pone.0329019 (PMC12312899; doi:10.1371/journal.pone.0329019)

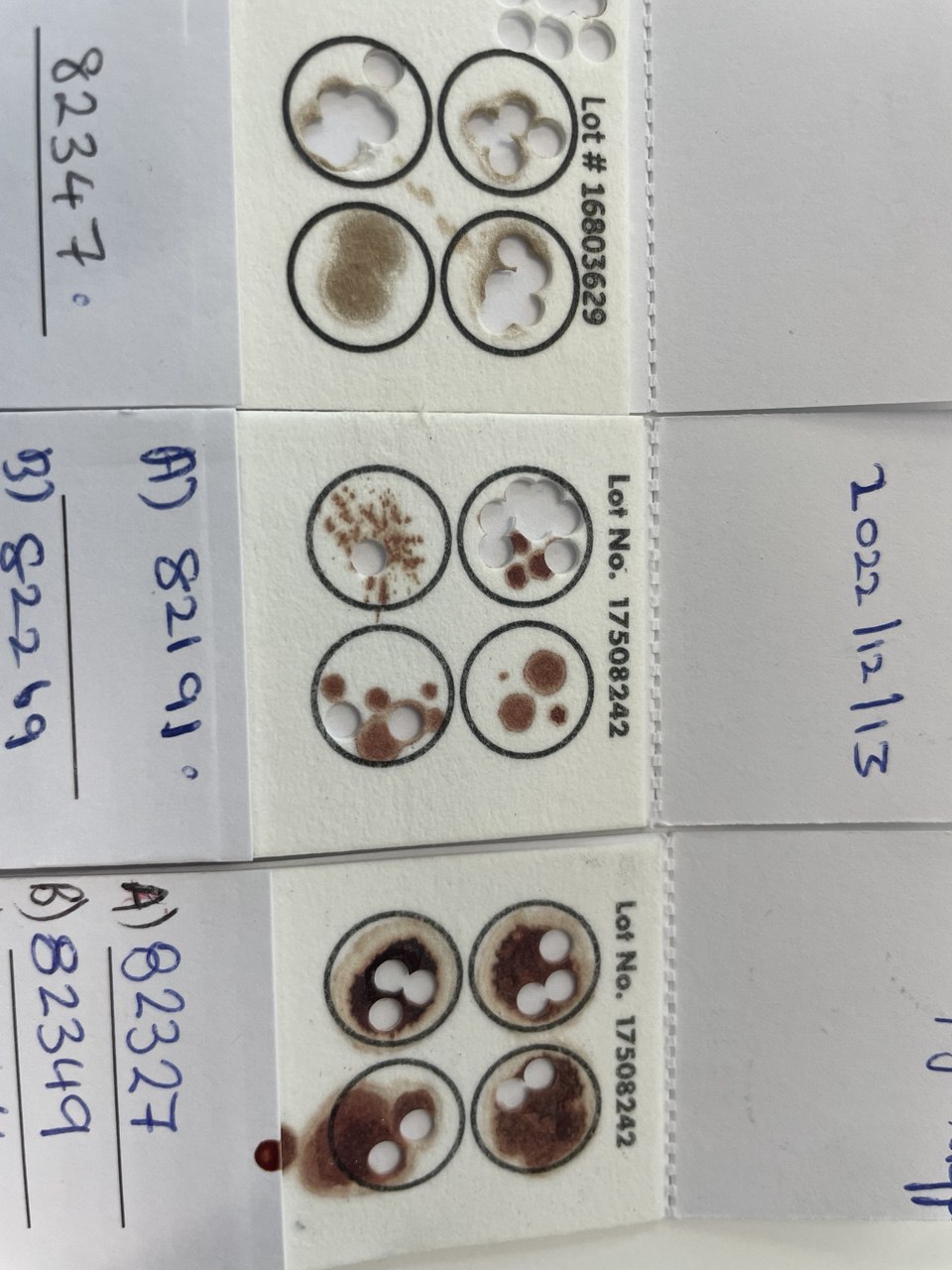

Supplement: S2 File — (JPG) [file pone.0329019.s002.jpg]

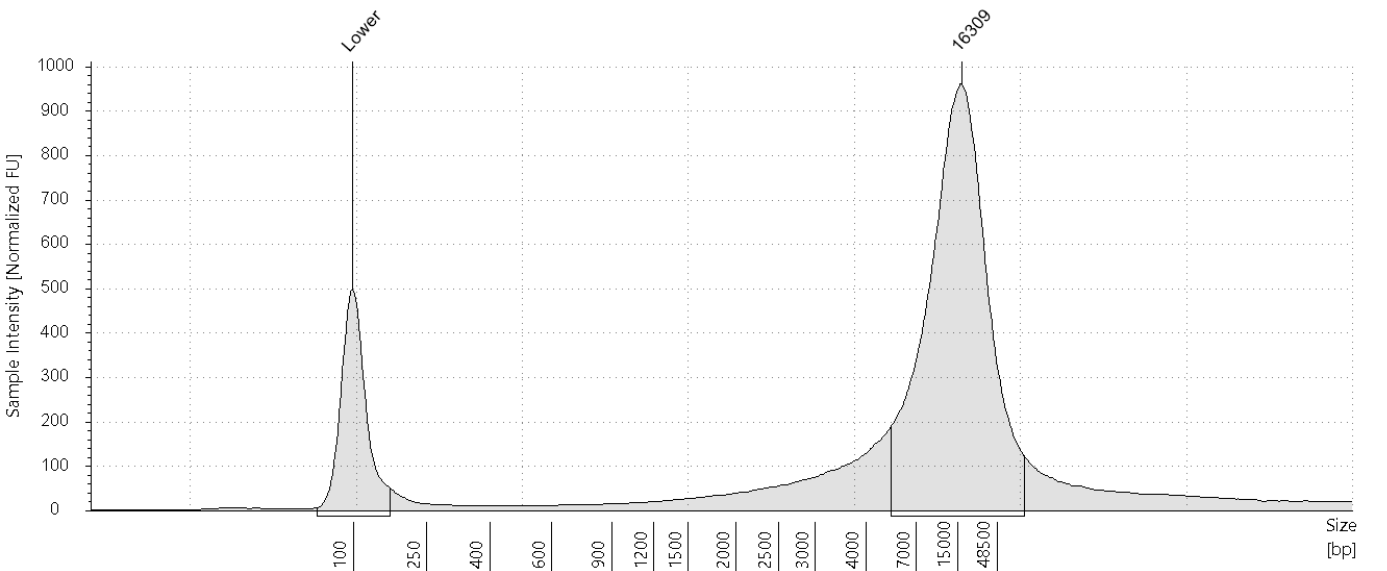

Supplement: S3 File — (ZIP) [file pone.0329019.s003.zip › S3/2024-05-03_-_14-44-21-gDNA_E1_82420.png]

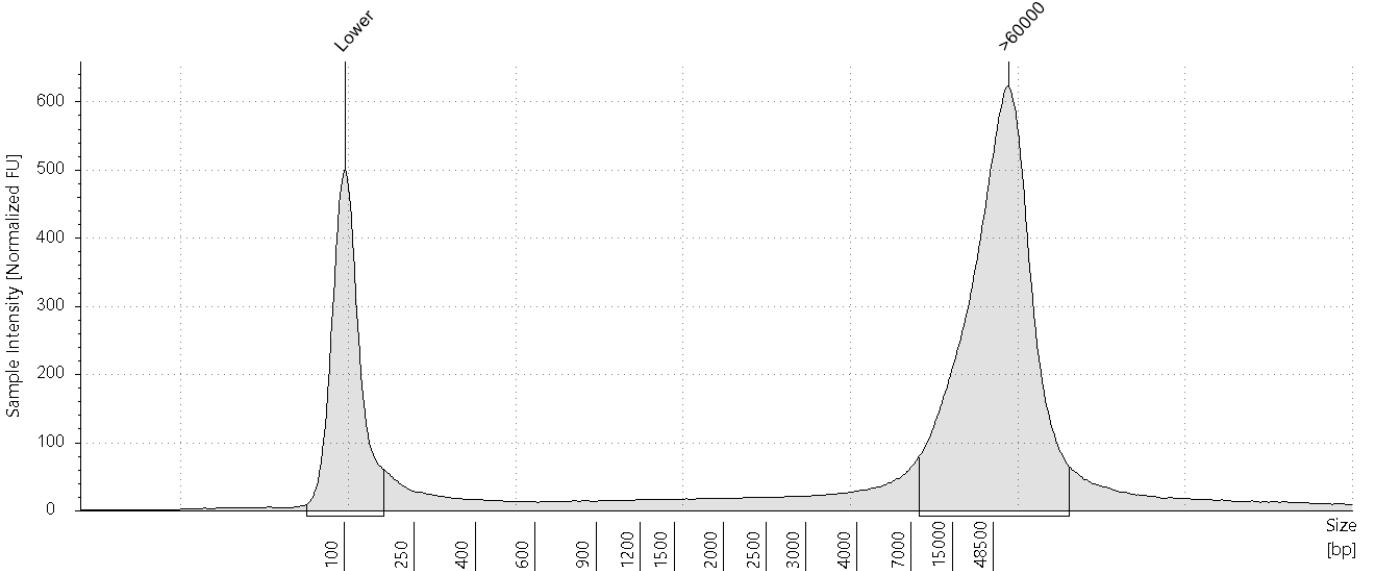

Supplement: S3 File — (ZIP) [file pone.0329019.s003.zip › S3/2024-04-12 - 10-45-31-gDNA_B1_42569.png]

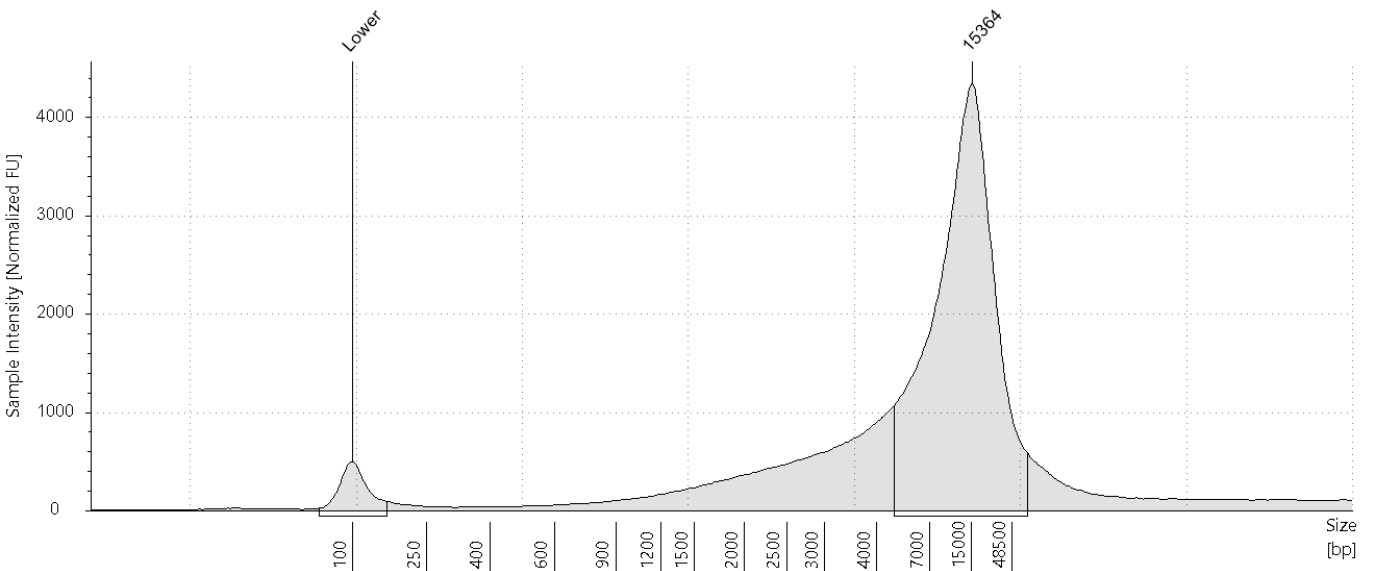

Supplement: S3 File — (ZIP) [file pone.0329019.s003.zip › S3/2024-04-03_-_13-03-55-gDNA_B1_2993.png]

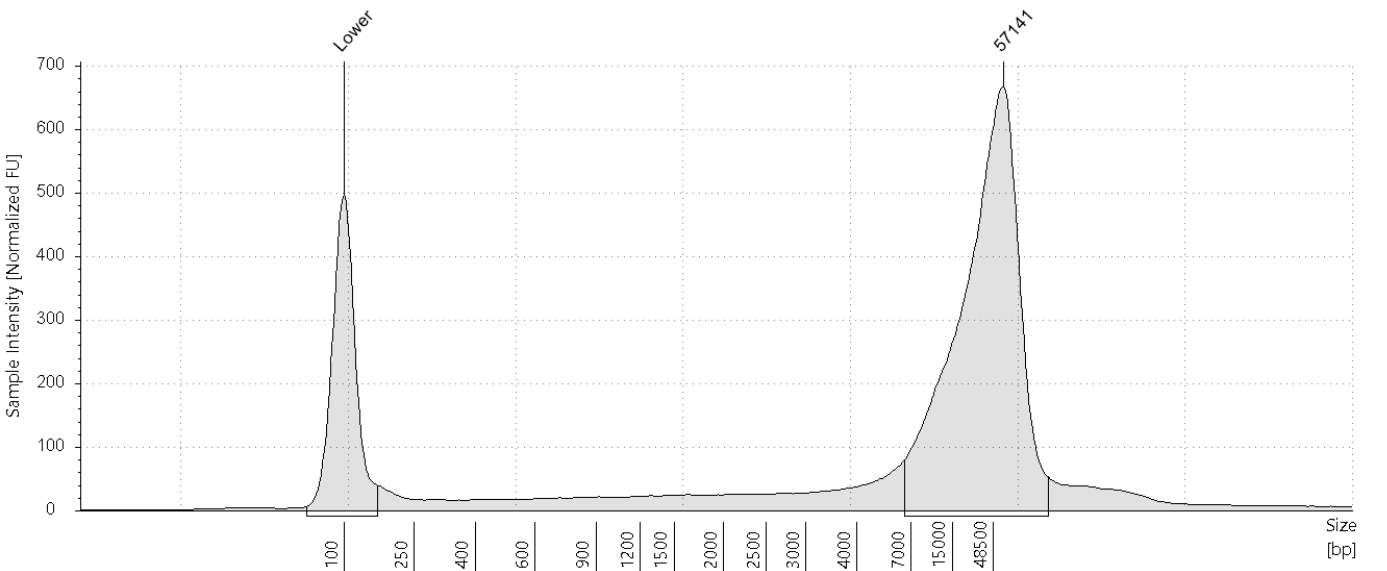

Supplement: S3 File — (ZIP) [file pone.0329019.s003.zip › S3/2024-04-12 - 10-45-31-gDNA_D1_42584.png]

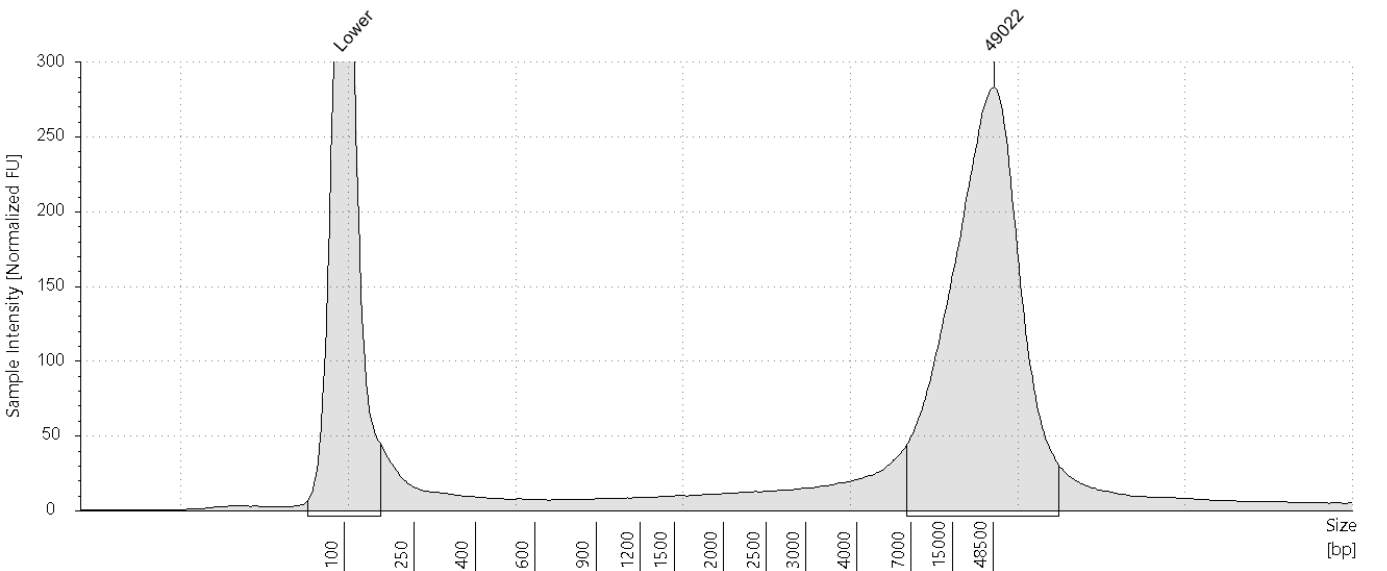

Supplement: S3 File — (ZIP) [file pone.0329019.s003.zip › S3/2024-04-12 - 10-45-31-gDNA_C1_49968.png]
